# Correction: Exposure of HL-60 human leukaemic cells to 4-hydroxynonenal promotes the formation of adduct(s) with α-enolase devoid of plasminogen binding activity

**DOI:** 10.1042/BCJ20090564_COR

**Published:** 2026-06-03

**Authors:** Giuseppina Barrera, Fabrizio Gentile, Stefania Pizzimenti, Alessia Arcaro, Piergiorgio Pettazzoni, Rosalba Minelli, Daniela D'Angelo, Gianfranco Mamone, Pasquale Ferranti, Cristina Toaldo, Gianpaolo Cetrangolo, Silvestro Formisano, Mario U. Dianzani, Koji Uchida, Chiara Dianzani

**Affiliations:** Dipartimento di Medicina e OncologiaSperimentale, Università di Torino, Corso Raffaello 30, Torino 10125, Italy

**Keywords:** α-enolase, 4-hydroxynonenal (4-HNE), 4-hydroxynonenal adducts, cell adhesion, HL-60 cells, plasminogen binding

The authors of the original article “Exposure of HL-60 human leukaemic cells to 4-hydroxynonenal promotes the formation of adduct(s) with α-enolase devoid of plasminogen binding activity” (DOI: 10.1042/BJ20090564) would like to correct the spelling of author ‘Gianpaolo CETRANGOLO’ to ‘Giovanni Paolo CETRANGOLO’.

The authors also request that the author contribution list is correctedand confirm that the authors listed first (Dr. Fabrizio Gentile), second (Dr. Stefania Pizzimenti), and third (Dr. Alessia Arcaro) contributed equally to this work as first co-authors.

The requested correction has been assessed and approved by the Editorial Board. The authors declare that these corrections do not change the results or conclusions of their paper.

